# Editorial: MicroRNAs as biomarkers in colorectal cancer

**DOI:** 10.3389/fonc.2022.1128392

**Published:** 2023-01-17

**Authors:** Paola Ulivi

**Affiliations:** Biosciences Laboratory, IRCCS Istituto Romagnolo per lo Studio dei Tumori (IRST) “Dino Amadori”, Meldola, Italy

**Keywords:** miRNA, liquid biopsy, colorectal, biomarker, prognostic

Colorectal cancer (CRC) is one of the most prevalent malignancies, known as the third most common cancer and a leading cause of global mortality. Despite advances in treatment and therapies for colorectal cancer patients, prognosis and overall survival rate remain poor. MicroRNAs (miRNAs) are small non-coding RNAs that modulate gene expression in both cells and tissues by targeting mRNA or inhibiting protein biosynthesis, therefore regulating cell growth and development. Studies have demonstrated the influence of miRNAs in the development of tumors and therefore are identified as potential diagnostic novel biomarkers for developing new therapies.

I would like to thank all the authors who contributed with their relevant work in the Research Topic “*MicroRNAs as biomarkers in colorectal cancer*.” miRNA expression, both in tumor tissue and in the blood, could give important diagnostic, prognostic, and predictive indications.

Guo et al., in their study, demonstrated that long non-coding RNA (lncRNA) miR31HG is upregulated in CRC tissue and is associated with a poor prognosis. Using *in vitro* and *in vivo* models, they demonstrated that the overexpression of MIR31HG is able to induce proliferation, growth, invasion, glycolysis, and lung metastases. Moreover, MIR31HG overexpression is able to induce YY1 messenger RNA (mRNA) and protein expression and inhibits miR-361-3p expression, which function as repressor of the malignant behavior of CRC cells. The authors concluded that MIR31HG acts as an oncogenic gene in CRC through a positive feedback loop of MIR31HG-miR-361-3p-YY1.

A role of miRNAs has also been demonstrated in relation to response to immunotherapy. Elevated levels of miR-425 and miR-576 are associated with tumorigenesis and a worse outcome (Hu et al.). Moreover, those miRNAs are negatively involved in PTEN-P53/transforming growth factor-β (TGF-β) immune function axis in CRC liver metastasis and are strongly associated with microsatellite instability, tumor microenvironment, and immune cell infiltration. This observation was inducted to the hypothesis that miR-425 and miR-576 could represent biomarkers for immunotherapy (Hu et al.). In another study by Huang et al., a signature of four miRNAs (miR-592, miR-625-3p, miR-552-5p, and miR-224-5p) was able to predict with high accuracy the tumor mutational burden status, suggesting their role in response to immune checkpoint inhibitors.

Liquid biopsy represents a valid strategy to evaluate and monitor miRNA expression. Cui et al., in their study, evaluated the expression of mir-1539 in exosomes and tissues of CRC patients and healthy controls, demonstrating a high diagnostic efficacy in discriminating between patients and healthy controls and a strong association with vascular endothelial growth factor expression. Moreover, at the tumor tissue level, miR-1539 expression was significantly associated with Ki-67 levels, suggesting a link of the miRNA with tumor progression and malignancy. In another study, Wei et al. analyzed the expression of miR-193a-5p in plasma-derived extracellular vesicles (EVs) of CRC patients and non-cancerous individuals. They demonstrated a downregulation of miR-193a-5p expression in cancer patients, confirming its role as tumor-suppressor miRNA. They demonstrated that miR-193a-5p inhibits CRC migration and invasion targeting tumor-associated genes like CUT-like homeobox 1 and intersectin 1. The plasma-EVs-miR-193a-5p could represent a promising biomarker for CRC detection.

## Author contributions

The author confirms being the sole contributor of this work and has approved it for publication.

